# Rhubarb Supplementation Prevents Diet-Induced Obesity and Diabetes in Association with Increased *Akkermansia muciniphila* in Mice

**DOI:** 10.3390/nu12102932

**Published:** 2020-09-24

**Authors:** Marion Régnier, Marialetizia Rastelli, Arianne Morissette, Francesco Suriano, Tiphaine Le Roy, Geneviève Pilon, Nathalie M Delzenne, André Marette, Matthias Van Hul, Patrice D Cani

**Affiliations:** 1 Metabolism and Nutrition Research Group, Louvain Drug Research Institute, Walloon Excellence in Life sciences and BIOtechnology (WELBIO), UCLouvain, Université catholique de Louvain, Av. E. Mounier, 73 B1.73.11, 1200 Bruxelles, Belgium; marion.regnier@uclouvain.be (M.R.); Marialetizia.Rastelli@uclouvain.be (M.R.); francesco.suriano@uclouvain.be (F.S.); tiphaine.le.roy@gmail.com (T.L.R.); nathalie.delzenne@uclouvain.be (N.M.D.); matthias.vanhul@uclouvain.be (M.V.H.); 2 Department of Medicine, Faculty of Medicine, Cardiology Axis of the Quebec Heart and Lung Institute, Quebec, QC G1V 4G5, Canada; arianne.morissette.2@ulaval.ca (A.M.); Genevieve.Pilon@criucpq.ulaval.ca (G.P.); Andre.Marette@criucpq.ulaval.ca (A.M.)

**Keywords:** Rhubarb, obesity, diabetes, gut microbiota, *Akkermansia muciniphila*, Reg3γ

## Abstract

Obesity and obesity-related disorders, such as type 2 diabetes have been progressively increasing worldwide and treatments have failed to counteract their progression. Growing evidence have demonstrated that gut microbiota is associated with the incidence of these pathologies. Hence, the identification of new nutritional compounds, able to improve health through a modulation of gut microbiota, is gaining interest. In this context, the aim of this study was to investigate the gut-driving effects of rhubarb extract in a context of diet-induced obesity and diabetes. Eight weeks old C57BL6/J male mice were fed a control diet (CTRL), a high fat and high sucrose diet (HFHS) or a HFHS diet supplemented with 0.3% (g/g) of rhubarb extract for eight weeks. Rhubarb supplementation fully prevented HFHS-induced obesity, diabetes, visceral adiposity, adipose tissue inflammation and liver triglyceride accumulation, without any modification in food intake. By combining sequencing and qPCR methods, we found that all these effects were associated with a blooming of *Akkermansia muciniphila*, which is strongly correlated with increased expression of *Reg3γ* in the colon. Our data showed that rhubarb supplementation is sufficient to protect against metabolic disorders induced by a diet rich in lipid and carbohydrates in association with a reciprocal interaction between *Akkermansia muciniphila* and Reg3γ.

## 1. Introduction

The prevalence of obesity has increased dramatically over the past several decades and continues to grow at pandemic rates [1,2,3]. The major cause of obesity resides in the imbalance between energy intake and energy expenditure, although complex interactions between behavior, environmental, genetic and physiological factors explain the heterogeneity in the development of obesity between individuals [4,5]. Obesity and, more broadly, metabolic syndrome (see review [6] for definition) are strongly associated with the development of metabolic and cardiovascular diseases such as insulin resistance, type 2 diabetes, fatty liver disease, hypertension and other comorbidities [7,8,9,10]. Although, the prevention of obesity by reducing food intake and/or increasing energy expenditure is to promoted, recent attempts at long-term weight loss or management have not been encouraging [11]. Considering the complexity of the obesity pandemic, considerable efforts are still needed to develop efficient strategies able to attenuate the burden on health care [12]. Developing specific foods that have promising intrinsic health benefits is a possible intervention that has been proposed.

Prebiotics fall into this category. A prebiotic is defined as “a substrate that is selectively utilized by host microorganisms, conferring a health benefit” [13,14]. This definition covers different food components, including several non-digestible carbohydrates (i.e., fibers) and plant extracts-derived polyphenols. The growing interest for prebiotics over the last decades stems from their ability to remodel the intestinal microbial community, which constitutes the one of the first sites of interaction between the host and its diet [15,16,17,18]. In a context of diet-induced obesity, prebiotic administration is able to alleviate metabolic endotoxemia (i.e., circulating LPS), gut barrier dysfunction, dyslipidemia and insulin-resistance through mechanisms, involving the modulation of specific bacteria [19,20,21,22,23,24,25]. Although, more than 100 different taxa are affected by prebiotics in rodents [19,25], *Bifidobacterium spp* and *Lactobacillus* are generally thought to be the main drivers of their beneficial effects [26,27,28]. However, recent evidence highlighted that some prebiotics also target Verrucomicrobia phylum and particularly *Akkermansia muciniphila* [19,25,29,30,31,32,33], which has confirmed health-promoting effects in both rodents and humans by different mechanisms, linked to the reinforcement of the gut barrier function [24,34,35,36]. Indeed, in order to efficiently maintain microbial homeostasis, the intestinal barrier is composed of a dense mucus barrier and includes antimicrobial peptides (AMP). These components are highly modulated by the diet. High fat diet (HFD) feeding leads to reduced mucus thickness and decreased expression of AMP, all contributing to HFD-induced gut fragility and associated disorders [24,37,38,39,40].

In this study, we were interested in the effects of a dry extract of rhubarb roots, which is derived from *Rheum palmatum* (also known as *Rheum officinale* or Chinese rhubarb). This anthraquinone-rich crude extract has a long history of herbal usage in traditional Chinese medicine for the treatment of gastrointestinal diseases. The *Rheum* genus encompasses several species that have been attributed antioxidant, anti-cancer, anti-inflammatory, anti-allergic and anti-bacterial effects [41,42,43,44,45,46,47,48]. Among them, *Rheum palmatum* may exert hepatoprotective effects [49]. Additionally, *Rheum palmatum* has recently been demonstrated to attenuate hepatic inflammation induced by acute alcohol intake by a mechanism involving a modulation of the gut microbiota [50]. However, the effects of rhubarb extract have never been investigated during obesity and prediabetes. Therefore, the present study aimed to characterize the microbiota-associated metabolic benefits of a rhubarb extract in mice fed a high-fat and high-sucrose diet (HFHS). We found that a 0.3% supplementation with rhubarb extract protects mice efficiently against obesity, glucose intolerance, adiposity and adipose tissue inflammation. These beneficial effects were associated with a gut microbiota remodeling favoring the expansion of *Akkermansia muciniphila*.

## 2. Materials and Methods 

### 2.1. Mice

Eight-week-old male C57BL/6J mice (Janvier, Le Genest-Saint-Isle, France) were co-housed in pairs under Specific and Opportunistic Pathogen Free conditions (SOPF) and in a controlled environment (temperature of 22 ± 2 °C, 12-h daylight cycle) with free access to food and water. Mice were acclimatized during one week with a control diet (D12450H; Research diet) and then randomly assigned to one of three dietary conditions (*n* = 9–10/group): (1) CTRL group, fed a control diet containing 10% calories from fat (D12450J; Research Diet; New Brunswick, NJ, USA); (2) HFHS group, fed an high-fat and high-sucrose diet (HFHS), containing 45% calories from fat and 35% calories from carbohydrates (D12451; Research diet; New Brunswick); or (3) RHUB group, fed an HFHS diet supplemented with 0.3% (grams extract/grams food) rhubarb extract (Ortis, Elsenborn, Belgium) mixed in the HFHS diet (prepared by Research Diet under controlled conditions). A dose of 0.3% has been chosen to allow direct comparison to the study [50], hich described the effects of rhubarb root extract on control diets [50]. The composition of rhubarb, used in this study, refers to the composition used previously by Neyrinck et al., [50]. The percentage of anthracenic derivatives (expressed as rhein) is 5.07 on dry matter. All mice were fed ad libitum all along the experiment. Body weight, food intake and water intake were recorded weekly for eight weeks. Body composition was assessed once a week by using a 7.5-MHz time-domain nuclear magnetic resonance (LF50 minispec; Bruker, Rheinstetten, Germany). Feces were harvested at the beginning (Day 0), after 4 weeks (Day 28), and at the end (Day 56) of the experiment. In the final week of the experiment, feces were collected for each cage by transferring the animals to clean cages for a period of 24 h. After this, feces were manually collected, dried overnight at 60 °C and weighted to assess the amount of feces secreted per day. Then energy content was measured on a C1 calorimeter from IKA. Per cage containing two animals, one mean value was considered for analysis.

All mouse experiments were approved by and performed in accordance with the guidelines of the local ethics committee. Housing conditions were specified by the Belgian Law of 29 May 2013, regarding the protection of laboratory animals (agreement number LA1230314).

### 2.2. Oral Glucose Tolerance Test

An oral glucose tolerance test (OGTT) was performed after 7 weeks of experiment as previously described [23]. Briefly, 6h-fasted mice were given an oral glucose load (2 g glucose/kg body weight, and blood glucose levels were recorded at different time points, 30 min before and 15, 30, 60, 90 and 120 min after glucose load. Glycaemia was measured with a glucometer (Accu check, Roche, Basel, Switzerland) on blood droplets collected from the tip of the tail vein.

### 2.3. Tissue Sampling

At the end of the experiment (week 8) and after 6h of fasting, all mice were anesthetized with isoflurane (Forene, Abbott, Queenborough, Kent, UK), and blood was sampled. After exsanguination, mice were euthanized by cervical dislocation. Liver, adipose tissues and different sections of the intestines were dissected, weighted and immersed in liquid nitrogen before long-term storage at −80 °C for further analysis.

### 2.4. Biochemical Analysis

To determine the plasma insulin concentration, blood was harvested from the tip of the tail vein using capillaries prior to glucose load (−30 min) and 15 min after glucose load. Plasma insulin concentration was measured using an ELISA kit (Mercodia, Uppsala, Sweden), according to the manufacturer’s instructions. Insulin resistance index was determined by multiplying the area under the curve of the blood glucose (−30 to 15 min) and plasma insulin (30 min and 15 min) [51].

Hepatic inflammatory markers levels (RANTES, TNF-α, IL-6, IFN-γ) were quantified in 50 µL of liver tissue lysates (100 mg of protein in PBS, containing 0.5% bovine albumin serum) using Bio-Plex Pro Assay Mouse Cytokine (Bio-Rad, Hercules, USA).

Lipid peroxidation was evaluated by measuring thiobarbituric acid reactive substances (TBARS) that are formed as fat-degradation by-products. After liver lysis in saline solution (NaCl), aldehydes from protein carbonyl groups, contained in the lysate, react with thiobarbituric acid (TBA) forming an aldehyde-TBA complex, which can be detected by spectrophotometry (520 and 535 nm). Protein concentration was measured by the Bradford method using BSA as standard.

### 2.5. Liver Lipid Quantification

Liver lipids were measured after extraction according to Folch method et al., [52], as previously described [53]. Briefly, approximatively 100 mg of liver tissue was grinded in 2 mL of CHCl3:MeOH (2:1) and then homogenized using an ultrasonic homogenizer. Lipids were extracted by adding 400 µL of 0.9% NaCl solution and vigorously shaking. After centrifugation, the lower lipidic phase was collected in a new glass tube and dried under nitrogen. Glass tubes were weighted before, and after, lipid extraction, in order to estimate the total lipid content. The dried residue was solubilized in 1.5 mL isopropanol. Liver triglyceride and cholesterol concentrations were measured using kits coupling an enzymatic reaction with spectrophotometric detection of the reaction end-products (Diasys Diagnostic and systems, Holzheim, Germany), according to the manufacturer’s instructions.

### 2.6. Citrate Synthase Activity Assay

Citrate synthase activity in the brown adipose tissue was assayed in approximatively 10 mg of brown adipose tissue lysed in 20 volumes of CelLytic MT Cell Lysis containing 1% (vol/vol) of protease inhibitor cocktail P8340 (Sigma, Saint-Louis, MO, USA) by bead-beating. The lysate was centrifuged at 10,000 *g* during 10 min at 4 °C two times in order to remove the lipids and the tissue debris. Tissue extract was diluted 1:10 in a 100mM phosphate buffer (pH 7.1) containing 10 mM 5,5′-dithiobis-(2-nitrobenzoic acid) (DTNB) and 30 mM acetyl-CoA. After the addition of 10 mM oxaloacetate, free coenzyme A produced from the condensation of acetyl-CoA and oxaloacetate was bound to DTNB, and resulting change in light absorbance detected spectrophotometrically at 412 nm was used to determine the activity of citrate synthase (µmol/mg/s).

### 2.7. Histological Analyses

Subcutaneous adipose tissue depots were fixed in 4% paraformaldehyde for 24 h at room temperature. Samples were then immersed in ethanol 100% before processing for paraffin embedding. To determine the adipocyte tissue diameter, paraffin sections of 5 µM were stained with hematoxylin and eosin. Images were obtained using a SCN400 slide scanner and digital Image Hub software 561 (Leica Biosystems, Wetzlar, Germany). Adipocyte diameter was determined using ImageJ (National institutes of health, Bethesda, MD, USA). F4/80 positive areas in the adipose tissue were randomly counted after immunostaining with F4/80 antibody (Ab6640, Abcam, Cambridge, UK). All histological observations were full blind analyzed by three individuals (S.G, M.V.H and M.R). At least 5 fields/mice were randomly selected and obtained using SCN400 slide scanner and digital image hub software (Leica Biosystems, Wetzlar, Germany).

### 2.8. RNA Preparation and Real-Time qPCR Analysis

Total RNA was prepared from tissues using TriPure reagent (Roche, Basel, Switzerland). Quantification and integrity analysis of total RNA were performed by analyzing 1 μL of each sample in an Agilent 2100 Bioanalyzer (Agilent RNA 6000 Nano Kit, Agilent, Santa Clara, CA, USA). cDNA was prepared by reverse transcription of 1 μg total RNA using a reverse transcription system kit (Promega, Madison, WI, USA). Real-time PCR was performed with the CFX Manager 3.1 software (Bio-Rad, Hercules, CA) using Mesa Fast qPCR (GoTaq qPCR Master Mix, Promega, Madison, WI, USA) for detection, according to the manufacturer’s instructions. RPL19 was chosen as the housekeeping gene. All samples were performed in duplicate, and data were analyzed according to the 2^−ΔΔCT^ method. The identity and purity of the amplified product were assessed by melting curve analysis at the end of amplification. The primer sequences for the targeted mouse genes are presented in Supplemental Table S2.

### 2.9. Gut Microbiota Analysis

Feces were sampled for gut microbiota analysis at 3 different time points: The beginning of the study (day 0), after 4 weeks (day 28) and at the end of the experiment (day 56). Genomic DNA was extracted using a QIAamp DNA Stool Mini Kit (Qiagen, Hilden, Germany), according to the manufacturer’s instructions, including a bead-beating step. qPCR was performed with the CFX96 Bio-Rad Real-Time PCR system and CFX Manager 3.1 software (Bio-Rad, Hercules, CA, USA). Primers used for qPCR amplification are listed in Supplemental Table S1. The cycle threshold (CT) of each sample was compared with a standard curve made by diluting genomic DNA isolated from a pure culture of a type strain (BCCM/LMG, Ghent, Belgium; DSMZ, Braunshweig, Germany).

### 2.10. Bacterial DNA Sequencing

Feces from day 56 were then used for sequencing analysis. Genomic DNA extracted from fecal content of the mice was diluted at 20 ng/µL and used as template for the amplification of the V4 region of the bacterial 16S rRNA gene with the primers 515F(GTGYCAGCMGCCGCGGTAA) and 806R (GGACTACNVGGGTWTCTAAT). High-throughput sequencing of purified amplicons were analyzed using Illumina MiSeq cartridge, according to the manufacturer instructions, using 30 amplification cycles with an annealing temperature of 65 °C. As MiSeq sequencing enables paired 300-bp reads, the ends of each read overlap and can be stitched together to generate extremely high-quality, full-length reads covering the entire V4 region. The quality of the run was checked internally using PhiX, and for further analysis, each pair-end sequence was assigned to its sample using the previously integrated index. The resulting reads were processed through FROGS pipeline implemented on a galaxy instance [54,55]. The sequences were de-replicated and clustered using swarm method with an aggregation distance equal to 3 for the clustering [56]. Chimeras were removed using the Vsearch tool [56]. Sequences were then filtered to keep clusters, also called operational taxonomic units (OTUs) present in at least 4 out of 30 samples and representing 0.00005% of all sequences. The taxonomic affiliations were performed using 16S SILVA database (Silva 138, Max Planck institute, Bremen, Germany). The average number of sequences per sample was 121,510 sequences. Alpha diversity (Chao1 and Simpson) and the Jacard-Binary metric were performed using FROGS. An metric multidimensional scaling (MDS) plot using the Jacard-Binary metric was created using R. Multivariate ANOVA statistical analysis with Adonis was performed to determine the statistical differences of microbial community among the different diet groups. This test used the Jacard-Binary dissimilarly matrix as the input and was performed over 9999 permutations. The resulting *p*-value is the result from the comparison of the diets after 56 days of experiment. Abundance of phyla and genera was calculated as percent abundance of OTUs present in the entire microbiota.

### 2.11. Statistical Analysis

Mouse data are expressed as the mean ± s.e.m (standard error of mean). Statistical analyses were performed using Graphpad Prism for Windows (version 8.00; graphpad software, San Diego, CA, USA). One-way or two-way analysis of variance (ANOVA) was performed, followed by appropriate post-hoc tests (Dunnett’s or Bonferroni, respectively) when differences were significant. Bacterial DNA sequencing was analyzed using Kruskal-Wallis test with Dunn’s multiple comparison test. A *p*-value < 0.05 was considered significant. For all analyses, exclusion decision was supported by the use of the Grubbs test for outlier detection.

## 3. Results

### 3.1. Rhubarb Root Extract Prevents Obesity and Fat Mass Accumulation in Diet-Induced Obese Mice

**Figure 1:**
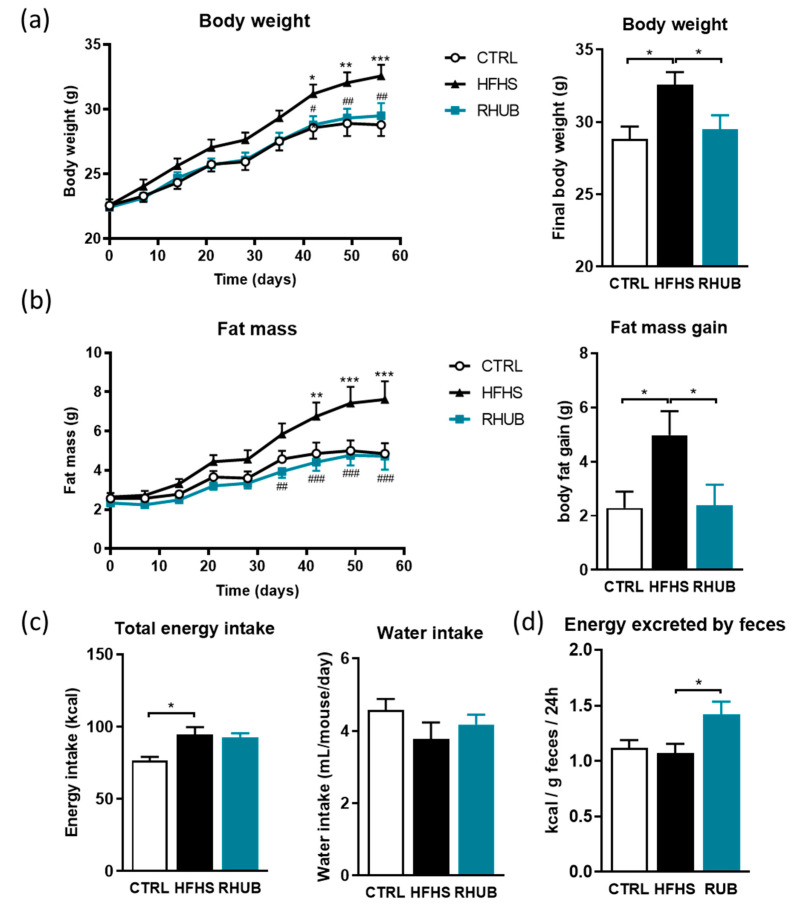
Rhubarb root extract prevents obesity and fat mass accumulation in diet-induced obese mice; (**a**) Body weight (g) over an 8 weeks period and final body weight; (**b**) Fat mass gain; (g) over an 8 weeks period and final fat mass gain; (**c**) Total energy intake (kcal) over the 8 weeks period and mean of the water intake (mL/mouse/day); (**d**) Energy measured in the feces at the end of the experiment (Kcal/g feces/24 h). White, CTRL fed mice; Black, HFHS fed mice and Blue, HFHS fed mice supplemented with rhubarb. Data represent mean ± SEM. * *p* ≤ 0.05, ** *p* ≤ 0.01, *** *p* ≤ 0.005. HFHS, High fat/high sucrose; RHUB, Rhubarb.

Mice were fed either a control diet (CTRL), a high fat and high sucrose diet (HFHS) or a HFHS diet supplemented with 0.3% (g/g) of rhubarb (RHUB) for 8 weeks. As expected, mice fed the HFHS diet gained more weight during the 8 weeks of follow-up compared to the ones fed the CTRL diet. However, rhubarb supplementation completely prevented this diet-induced weight gain and fat mass accumulation (Figure 1a,b). At the end of experiment, mice supplemented with rhubarb (RHUB) exhibited body weights and adiposities similar to that of control-diet fed mice (CTRL) that differed significantly from that of the HFHS-group from day 42 on (Figure 1a,b). Food intake was not affected by rhubarb-supplementation and both HFHS and RHUB groups showed increased caloric intake. This suggests that protective effects of rhubarb supplementation on weight gain and fat mass accumulation could not be attributed to a reduction in energy intake (Figure 1c). However, we found that rhubarb supplementation was associated with a significantly increased fecal energy excretion (Figure 1d).

### 3.2. Rhubarb Root Extract Blunts Glucose Intolerance, Hepatic Steatosis and Liver Inflammation

**Figure 2:**
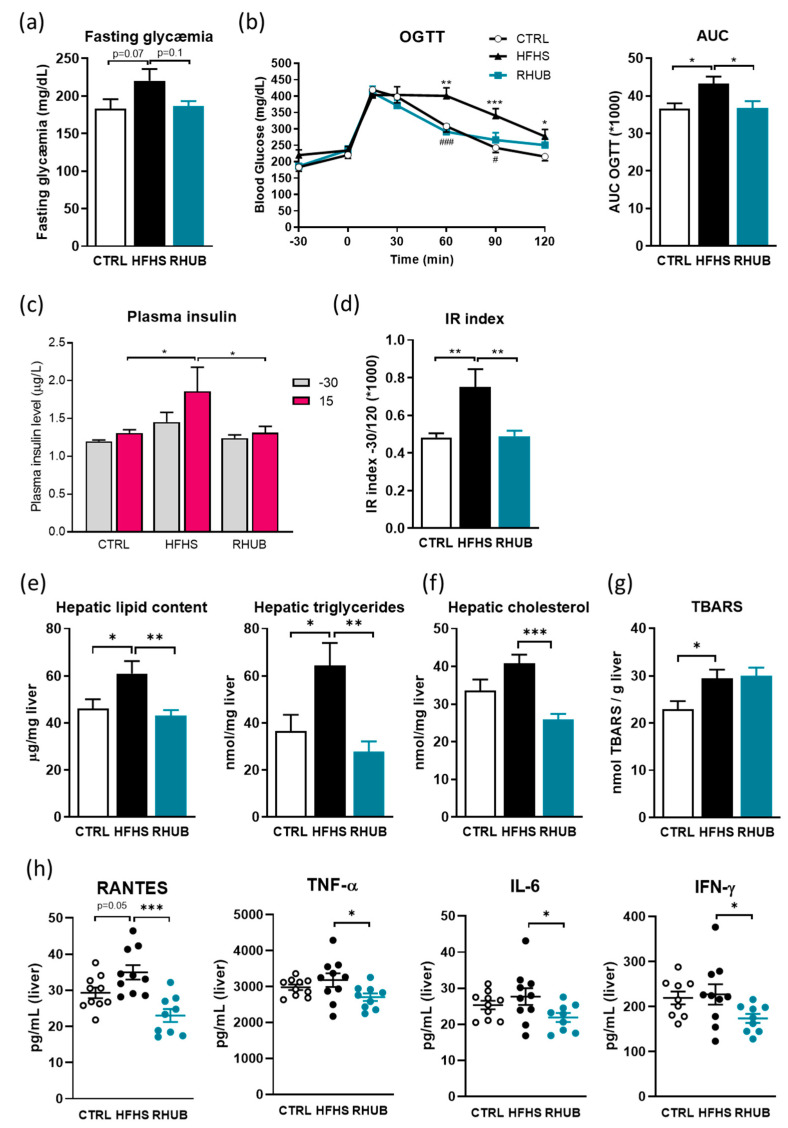
Rhubarb root extract blunts glucose intolerance, hepatic steatosis and liver inflammation (**a**) Plasmatic glucose (mg/dL) measured 6 h after a fasting period. (**b**) Plasma glucose profile (mg/dL) measured between 30min before and 120 min after glucose loading (*n*  = 8–10/group) and corresponding area under the curve. (**c**) Plasma insulin levels (µg l−1) at 30 min before and 15 min after glucose loading. (**d**) Insulin resistance index determined by multiplying the AUC of blood glucose by the AUC of insulin. (**e**) Hepatic lipid content (µg lipids/mg liver) and hepatic triglycerides (nmol/mg liver) measured by Folch. (**f**) Hepatic cholesterol (nmol/mg liver) measured by Folch. (**g**) Thiobarbituric acid reactive substances (TBARS) in the liver. (**h**) Hepatic inflammation markers measured using Bioplex. White, CTRL fed mice; Black, HFHS fed mice and Blue, HFHS fed mice supplemented with rhubarb. Data represent mean ± SEM. * *p* ≤ 0.05, ** *p* ≤ 0.01, *** *p* ≤ 0.005. OGTT, Oral glucose tolerance test; AUC, Area under the curve; IR Index, Insulin resistance index; TBARS, Thiobarbituric acid reactive substances; RANTES, Regulated upon Activation, Normal T Cell Expressed and Presumably Secreted; TNF-α, Tumor necrosis factor-alpha; IL-6, Interleukin-6; IFN-γ, Interferon-gamma.

In response to the HFHS diet, mice developed a mild fasting hyperglycemia and glucose intolerance, both defining prediabetes (Figure 2a,b). In contrast, supplementation with the rhubarb extract completely abolished these effects. This was concomitant with an improvement in glucose load-induced hyperinsulinemia (Figure 2c), resulting in a significant reduction of the insulin resistance index (Figure 2d). Liver weight was unaffected in rhubarb-treated mice (data not shown), but diet-induced liver steatosis (determined by measuring hepatic lipid content and hepatic triglycerides) was prevented by rhubarb supplementation (Figure 2e). Hepatic cholesterol levels were also significantly reduced in rhubarb-treated mice (Figure 2f). This was associated with a significant decrease in hepatic levels of inflammatory markers such as RANTES, TNF-α, IL-6 and IFN-γ (Figure 2h). The level of thiobarbituric acid reactive substances (TBARS) remained unchanged compared to HFHS fed mice, suggesting that rhubarb supplementation alleviated hepatic inflammation but not oxidative stress (Figure 2g). Taken together, these data show that rhubarb supplementation effectively affects glucose homeostasis and liver metabolism and prevents prediabetes, insulin resistance and liver steatosis in a mouse model of diet-induced obesity.

### 3.3. Rhubarb Root Extract Protects Against Adiposity and Adipose Tissue Inflammation

**Figure 3:**
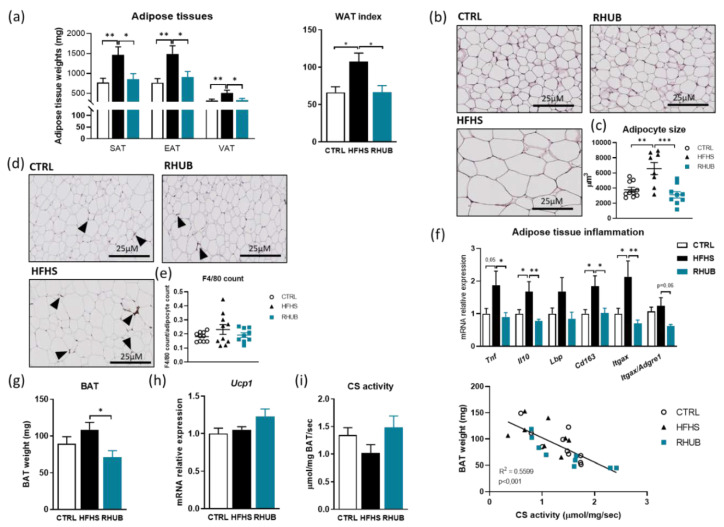
Rhubarb root extract protects against adiposity and adipose tissue inflammation (**a**) Adipose tissue weights (mg) measured after 8 weeks of experiment. SAT, subcutaneous adipose tissue, EAT, epididymal adipose tissue, VAT, visceral adipose tissue. White adipose tissue index measuring by adding all adipose tissue weights. (**b**) Representative adipose tissue Hematoxylin and Eosin staining. Scale bar, 25 µM. (**c**) Adipocyte size (µm^3^). (**d**) Representative adipose tissue F4/80 immunohistological staining. Scale bar, 25 µM. (**e**) Counting of the F4/80 positive area. (**f**) mRNA expression of different inflammatory markers in the adipose tissue. (**g**) Brown adipose tissue weight (mg) measured after 8 weeks of experiment. (**h**) mRNA expression of *Ucp1* in the brown adipose tissue. (**i**) Brown adipose tissue citrate synthase activity (µmol/mg BAT/sec) and correlation between brown adipose tissue weight and citrate synthase activity. *p*-value and R^2^ come from linear regression. White, CTRL fed mice; Black, HFHS fed mice and Blue, HFHS fed mice supplemented with rhubarb. Data represent mean ± SEM. * *p* ≤ 0.05, ** *p* ≤ 0.01, *** *p* ≤ 0.005. WAT, White adipose tissue, BAT, Brown adipose tissue, CS, citrate synthase.

As expected, mice fed a HFHS diet accumulated significantly more fat mass during the 8-week follow-up than the control mice. However, mice for which the HFHS diet was supplemented with rhubarb root extract were resistant to excessive fat mass accumulation, and had fat pads (visceral, epididymal and subcutaneous) of similar proportions as those of the control mice (Figure 3a). Adipocyte morphology from rhubarb-supplemented mice was similar to CTRL treated animals (Figure 3b,c). Next, we performed an immunohistological F4/80 staining, in order to visualize macrophages infiltration in the subcutaneous fat depots. Histological pictures showed that rhubarb-treated mice exhibited a trend towards decrease in macrophage infiltration (Figure 3d,e). In line with these results, we found that rhubarb exerts anti-inflammatory effects in the adipose tissue. The mRNA expression of inflammatory markers *Tnf*, *Il10*, *Lbp* and *Itgax* were significantly decreased after rhubarb supplementation (Figure 3f). Rhubarb-treated mice also had lower brown adipose tissue (BAT) weight (Figure 3g) and this was associated with increased *Ucp1* mRNA expression (Figure 3h), although this did not reach the significance threshold. To assess activity and number of mitochondria in the BAT, we measured the citrate synthase activity and found that rhubarb increased the activity of citrate synthase and that this is strongly correlated with BAT weight (*p* < 0.001) (Figure 3i). Taken together, these data suggest that the rhubarb root extract prevents diet-induced adiposity and adipose tissue inflammation.

### 3.4. Rhubarb Root Extract Affects Antimicrobial Peptides and Intestinal Renewal

**Figure 4:**
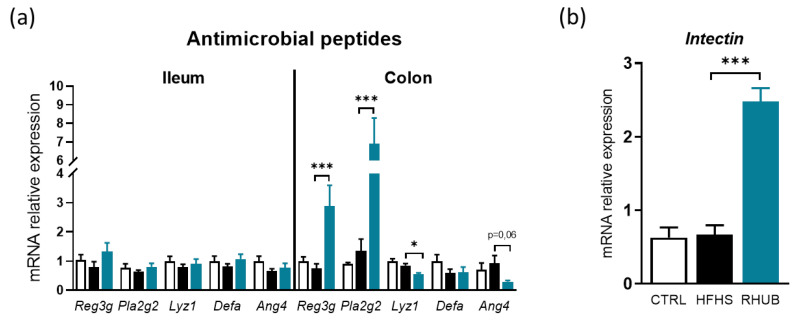
Rhubarb root extract affects antimicrobial peptides and intestinal renewal (**a**) mRNA expression of different antimicrobial peptides in the ileum (left panel) and in the colon (right panel). (**b**) mRNA expression of intectin in the colon. White, CTRL fed mice; Black, HFHS fed mice and Blue, HFHS fed mice supplemented with rhubarb. Data represent mean ± SEM. * *p* ≤ 0.05, ** *p* ≤ 0.01, *** *p* ≤ 0.005.

Next, we investigated whether the observed metabolic benefits were associated with an improvement in key markers of the gut barrier function. We previously discovered that prebiotics, such as inulin-type fructans maintain intestinal microbiota homeostasis by acting on several lines of defense of the gut barrier, such as the production antimicrobial peptides (AMP), and intestinal epithelial cells renewal [25]. We did not observe a specific alteration of several AMP in HFHS treated mice (Figure 4a). However, we found that rhubarb increased the mRNA expression of *Reg3g* and *Pla2g2* by ~3 and ~7 fold, respectively in the colon, suggesting that rhubarb reinforces innate immunity to maintain mucosal homeostasis (Figure 4a). The mRNA expression of other AMP such as *Lyz1* and *Ang4* decreased in mice supplemented with rhubarb (Figure 4a). In addition, mRNA expression of *Intectin*, a key protein involved in intestinal epithelial cell turnover was strongly induced (~2.5 fold) in rhubarb fed mice (Figure 4b). These findings suggest that the rhubarb root extract is able to reinforce gut barrier integrity by targeting specific antimicrobial peptides, and by increasing epithelial cell renewal.

### 3.5. Effects of Rhubarb Root Extract on Gut Microbiota Composition

**Figure 5:**
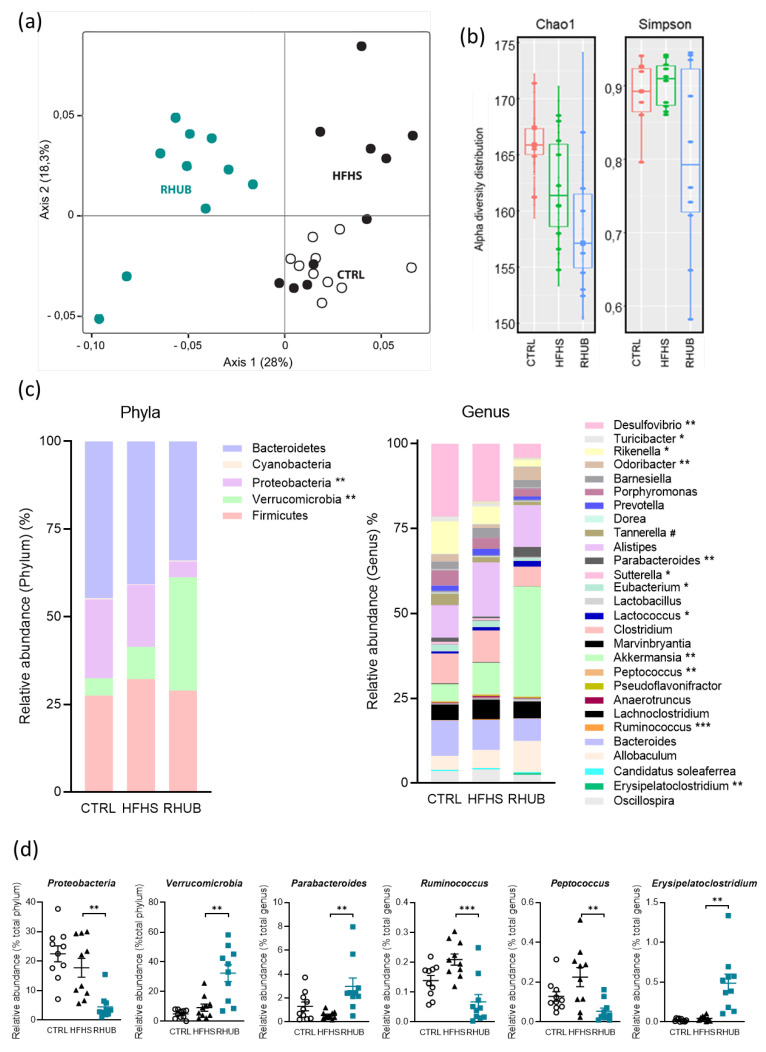
Effects of rhubarb root extract on gut microbiota composition (**a**) Non-metric multidimensional scaling (MDS) representing the Jaccard-binary differences between individuals within each group. (**b**) Bacterial α-diversity from fecal microbiota. Bacterial richness (Chao1 index) is shown on the left; bacterial evenness (Simpson index) is shown on the right. (**c**) Relative abundance obtained by OTUs of the major bacterial phyla (left) and genera (right). (**d**) Relative abundance of specific bacterial phyla and genera in each sample among the CTRL, HFHS and RHUB group. (**c**,**d**) Kruskal-Wallis test with Dunn’s multiple comparison test. * *p* ≤ 0.05, ** *p* ≤ 0.01, *** *p* ≤ 0.005 for HFHS vs. RHUB; ^#^ *p* ≤ 0.05, ^##^ *p* ≤ 0.01, ^###^ *p* ≤ 0.005 for CTRL vs. HFHS.

Then, we analyzed the gut microbiota of mice from CTRL, HFHS and RHUB groups by using metagenomics analysis. At day 56, three distinct microbial communities are observed by the non-metric multidimensional scaling (MDS) (ANOVA performed with Adonis, F.Model = 5.5856, *p* < 0.0001). In line with our previous observations, MDS revealed that rhubarb root extract strongly impacts on gut microbiota composition (first axis) and this effect is superior to the one of HFHS (second axis) (Figure 5a). Interestingly, rhubarb-fed group clustered differently from HFHS group but also from CTRL mice, suggesting that rhubarb may remodel the gut microbiota independently from the diet. Then, we investigated the effect of rhubarb supplementation on alpha-diversity distribution. Chao1 and Simpson indexes showed that rhubarb-fed mice exhibited a reduced richness (*p* = 0.00156) and evenness (*p* = 0.0149), respectively (Figure 5b). Then, we deeply analyzed the composition of gut microbiota by examining phylum and genus microbial changes among the groups. Interestingly, while the Firmicutes/Bacteroidetes ratio is almost unchanged, rhubarb-fed mice exhibited a large increase in Verrucomicrobia and a decrease in Proteobacteria phyla (Figure 5c,d). Analyses at genus level confirmed that rhubarb root extract drastically changed the composition of gut microbiota since 12 out of 28 bacterial genera are significantly affected by rhubarb supplementation (Figure 5c). Among the most affected genera, the abundance of *Akkermansia muciniphila, Parabacteroides* and *Erysipelatoclostridium* significantly increased upon rhubarb supplementation, while *Ruminococcus* and *Peptococcus* significantly decreased in rhubarb-fed mice. Therefore, we found that rhubarb root extract massively and specifically remodeled the gut microbiota, with a stronger impact on some specific genera.

### 3.6. Rhubarb Root Extract Promotes the Growth of Akkermansia muciniphila in HFHS Fed Mice

**Figure 6:**
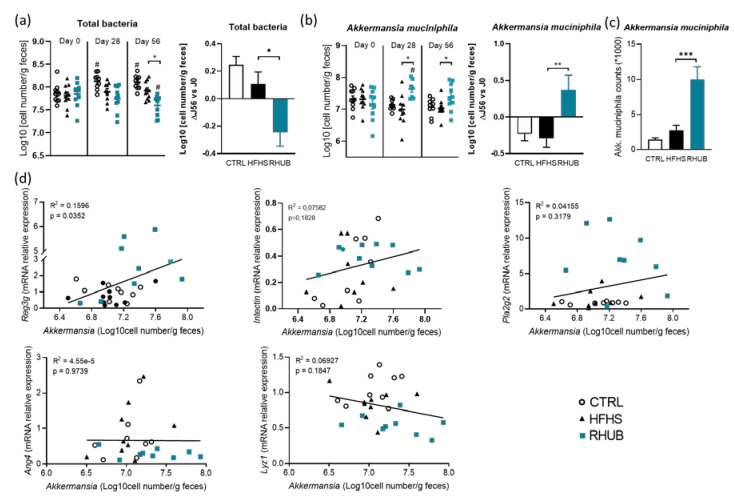
Rhubarb root extract promotes the growth of *Akkermansia muciniphila* in response to HFHS (**a**) Total bacteria measured by qPCR at day 0, day 28 and day 58 of experiment (Left panel). Difference of total bacteria between day 56 and day 0 (right panel). (**b**) qPCR analysis of *Akkermansia muciniphila* in the fecal content (left panel). Difference in *Akkermansia muciniphila* between day 0 and day 56 (right panel). (**c**) Relative abundance (percentage of 16S rRNA gene sequences) of *Akkermansia muciniphila* measured in fecal content of mice after 56 days of experimentation. (**d**) Correlation between *Akkermansia muciniphila* and *Reg3y*, *Pla2g2*, *Intectin*, *Ang4* and *Lyz1* mRNA expression, respectively. The *p*-value and R^2^ come from linear regression. White, CTRL fed mice; Black, HFHS fed mice and Blue, HFHS fed mice supplemented with rhubarb. Data represent mean ± SEM. * *p* ≤ 0.05, ** *p* ≤ 0.01, *** *p* ≤ 0.005. R², Coefficient of determination.

Given that the most affected phylum and genus after sequencing were Verrucomicrobia and its unique representative in the gut, *Akkermansia muciniphila*, we were interested in precisely quantifying *Akkermansia muciniphila* by qPCR. Indeed, its abundance is known to be increased by some prebiotics [19,25,29,30,31,32,33], and we have previously linked this genus to the improvements of intestinal barrier, obesity and diabetes [24,34,35,36]. Given that we have previously observed a link between specific bacteria, such as *Akkermansia muciniphila* and the expression of several antimicrobial peptides (AMP), we specifically followed the evolution of *Akkermansia muciniphila* abundance from the beginning to the end of the experiment. Fecal samples were harvested after acclimatization time (Day 0), after 4 weeks of CTRL, HFHS or HFHS-RHUB diet exposition (Day 28) and at the end of the study (Day 56). At baseline (Day 0), the similar bacterial load between all groups was confirmed by measuring total bacteria by qPCR. In contrast, the quantity of fecal bacteria differed progressively from day 28 to day 56, according to diet composition (Figure 6a). Mice fed the CTRL diet showed increased number of total bacteria starting from day 28. Rhubarb supplementation, on the other hand, led to a decrease in total amount of bacteria by Day 56, resulting in a negative delta between the beginning and the end of the experiment, thereby validating the results from metagenomics analysis (Figure 6b). These findings confirm previous results, obtained after 17 days of rhubarb exposure [50]. Next, we investigated the abundance of *Akkermansia muciniphila*, known to be modulated by HFHS and prebiotic exposures [26,27,28]. Interestingly, we found that, while the abundance of *Akkermansia muciniphila* tended to decrease in CTRL- and HFHS-exposed mice, rhubarb supplementation significantly increased the abundance of *Akkermansia muciniphila* from day 28 (Figure 6b). The difference in abundance between day 0 and day 56 was positive only for the rhubarb-supplemented mice (Figure 6b). Moreover, metagenomics analysis revealed that rhubarb-fed mice exhibited massive increase in the relative abundance of *Akkermansia muciniphila* (Figure 5c and Figure 6c). When investigating whether changes in *Akkermansia muciniphila* composition were correlated with immunity, we found that while *Akkermansia muciniphila* is poorly associated with *Pla2g2*, *Lyz1*, *Defa*, *Ang4* and *Intectin* mRNA expression in the colon, a positive correlation was observed between *Reg3y* gene expression and the abundance of *Akkermansia muciniphila* (Figure 6d). Interestingly, the rhubarb-fed mice who exhibited the lowest levels of *Akkermansia muciniphila* at the end of the experiment (qPCR and 16S sequencing) also showed the lowest mRNA expression of *Reg3y*. These findings suggest that one mechanism by which the rhubarb root extract could preserve intestinal barrier integrity is by reinforcing the crosstalk between *Akkermansia muciniphila* and Reg3y.

## 4. Discussion

There is a large body of evidence supporting the beneficial effects of plant-derived extracts and fibers on health, and particularly on the features of metabolic syndrome. However, the underlying mechanisms often remain elusive.

In this study, we assessed the properties of a polyphenol-rich rhubarb extract on the metabolic consequences in mice fed a high-fat, high-sucrose diet, which closely mimics the unhealthy human dietary pattern that leads to obesity-associated disorders [57]. We found that rhubarb supplementation (0.3 g/100 g diet that is equivalent to 8.7 mg/mice/day or 294.2 mg/kg body weight for mice and a human equivalent dose = 23.5 mg/kg) efficiently prevented diet-induced obesity, visceral adiposity, glucose intolerance, hepatic steatosis and adipose tissue inflammation induced by the HFHS diet. The overall health improvements were associated with a decrease in the expression of inflammatory genes and a lower number of macrophages in the adipose tissue, showing the anti-inflammatory potency of the rhubarb extract also in the context of obesity and glucose intolerance. 

The anti-obesogenic effect of rhubarb is likely related to an increase of energy excretion/expenditure rather than to a decrease in energy intake. Indeed, we did not observe any difference in food intake, but we found; (1) an increase in the energy excreted via the feces; and (2) an increased mitochondrial activity evidenced by increased citrate synthase activity. Despite their relatively low abundance compared to white adipocytes, brown adipocytes are clinically relevant because they exhibit high metabolic activity in response to several stimuli (cold, ketogenic diet, β-adrenergic stimulation…) and this adaptive thermogenesis is pertinent in the context of obesity [58,59,60,61,62]. Recently, an increase in BAT activity has been identified as one of the mechanisms protecting mice from obesity in mice treated with an extract of camu camu, a polyphenol-rich berry [63].

We and others have found in rodents that specific prebiotics, such as inulin-type fructans and some polyphenols specifically protected mice from obesity and diabetes-associated glucose metabolic disturbances, linked to an increase in bacterial diversity [19,20,21,22,23,24,25]. Here, we found that HFHS-fed mice have lower fecal alpha-diversity levels. Surprisingly, this reduction was intensified in rhubarb-supplemented mice. These results confirm the findings from a previous study showing that the decrease in alpha-diversity in response to rhubarb supplementation was due to a lower evenness, rather than a decrease in the bacterial richness [50]. The observed metabolic effects of prebiotics have previously been linked with changes in the microbiota and are often associated with an increased abundancy of *Akkermansia muciniphila*, a commensal bacterium with proven health-promoting properties [19,24,25,29,30,31,32,33,34,35,36]. Moreover, several herbal-based dietary intervention studies have been shown to increase the abundance of *Akkermansia muciniphila* [64,65].

Here, we found that although the rhubarb extract acted on the overall gut microbiota composition, the major impact was observed on the phylum Verrucomicrobia, which is represented by the genus *Akkermansia muciniphila*. This effect on *Akkermansia muciniphila* had already been described for certain rhubarb extracts under standard diet and diet-induced obesity and diabetes [50,66,67]. Under control diet, rhubarb extract supplementation led to an increase in the abundance of *Akkermansia muciniphila,* which was associated with metabolic improvements (increased crypt depth and mRNA expression of antimicrobial peptides) [50]. Under diet-induced obesity, high doses of a purified anthraquinone-glycoside preparation from rhubarb (400 mg/kg) or Sennoside A (an active ingredient of rhubarb) increased *Akkermansia muciniphila* and were as efficient as metformin on glucose tolerance, obesity and inflammation [66,67]. However, the mechanism of action of rhubarb extracts against obesity remain unclear. Although, we cannot directly attribute the rhubarb-induced metabolic improvements to the blooming of *Akkermansia muciniphila*, several indications imply that *Akkermansia muciniphila* might be involved.

First, the rhubarb crude extract is rich in polyphenols, particularly in anthraquinones, which represent the main bioactive ingredients of rhubarb, and have been described as anti-inflammatory, anti-tumoral and hepatoprotective agents [68] (see Supplemental Figure S1). Polyphenols are poorly digested in the upper intestinal tract and, as a consequence, reach the colon intact, where they can be metabolized by specific bacteria [69] and impact directly on gut microbiota composition.

Second, *Akkermansia muciniphila* has previously been shown to reverse HFD-induced obesity and diabetes by affecting metabolism and improving gut barrier function [24,36,70]. These findings have been supported by a recent clinical study demonstrating that *Akkermansia muciniphila* ameliorates several metabolic parameters in human (i.e., insulin resistance, cholesterol and markers of hepatic and systemic inflammation) [34]. Moreover, this genus has been demonstrated to be positively regulated by polyphenols in both physiological and pathological conditions [29,30,31,32,33,71,72,73]. Therefore, we speculate that *Akkermansia muciniphila* is involved, and perhaps even required, in achieving the metabolic improvements induced by rhubarb supplementation.

Although, we propose that *Akkermansia muciniphila* is likely involved in the beneficial effects of the rhubarb extract, the mechanisms behind may have different origins. In reality, rhubarb might directly interact with goblet cells producing mucus to modulate the production of *Akkermansia muciniphila*. It is accepted that *Akkermansia muciniphila* uses intestinal mucins (proteins of the epithelial mucus layer) as its main source of carbon and nitrogen [74]. Moreover, some phenolic fractions such as proanthocyanidins modulate the abundance of *Akkermansia muciniphila,* while concomitantly expanding mucus thickness [75]. However, we observed no significative change in the expression of *Muc2*, one of the members of mucin protein family, suggesting that rhubarb exerts its prebiotic effect on *Akkermansia muciniphila* independent of mucus secretion (data not shown).

Another mechanism might depend on the antimicrobial peptides (AMP), also known as host defense peptides, which are secreted by the epithelial cells covering the mucosal surface (Paneth cells). Most AMP have direct antimicrobial activities, whereas others act indirectly by modulating the host defense systems [76]. They form an important component of the gut ecosystem and participate to the microbial adaptation upon diet-induced obesity [37,38,39]. The best-characterized AMP are Reg3γ, Pla2g2, Lys1, Defa and Ang4. These peptides act as a firewall against mucin-degrading species by affecting their viability [77]. We found that rhubarb supplementation led to huge increases in the expression of two critical AMP, *Reg3-γ* and *Pla2g2*. *Akkermansia muciniphila* had previously been positively correlated with intestinal expression of *Reg3γ*, a C-type lectin that specifically targets Gram-positive bacteria [24,25], a finding that we confirmed in this study. It is interesting to note that “Reg3-γ non-responders” (mice that had very low expression of *Reg3-γ*), also had the lowest fecal levels of *Akkermansia muciniphila*, suggesting that gut microbiota composition and especially *Akkermansia muciniphila* determined the levels of Reg3-γ, and thereby participated in improvement in gut barrier function in response to rhubarb supplementation. We cannot exclude that other bacteria are involved in the regulation of AMP, especially because *Reg3-γ* expression is also well-correlated with the increase in *Bifidobacterium* in mice fed an HFHS diet supplemented with rhubarb (data not shown). Therefore, we hypothesized that rhubarb might prevent the overgrowth of pathogenic bacteria by stimulating the crosstalk between *Akkermansia muciniphila* and *Reg3γ*, thereby participating to protect against HFHS-induced disorders.

Other intestinal agents are also involved in the regulation of gut barrier integrity. Intectin is a glycosylphosphatidyl inositol-anchored protein that is a specific resident of the small intestine. Intectin is localized on the mucosal villi, where it facilitates the rapid turnover of intestinal mucosa [78]. Here, we found that rhubarb increased the colonic expression of *Intectin* more than two-fold. However, this was not correlated with *Akkermansia muciniphila* abundance. Up-regulation in the mRNA expression of *Intectin* has already been demonstrated with inulin-type fibers but never with polyphenol-rich prebiotics [25]. Recently, we demonstrated that administration of *Akkermansia muciniphila* in mice attenuated the down-regulation of *Intectin* that occurred, in response to HFD [79]. This represents another mechanism by which rhubarb extract supplementation can contribute to reinforce the gut barrier function [25,80,81]. Moreover, the increased expression of *Intectin* is accompanied by an increase in fecal energy excretion. This led us to postulate that an increase in epithelial cell renewal may contribute, not only to maintaining barrier integrity, but also to higher energy elimination via the feces. Increased fecal energy excretion could therefore represent a potential mechanism mediating the effects of rhubarb.

In summary, this study unveiled the beneficial properties of a rhubarb crude extract in a murine model of diet-induced obesity using a high-fat/high-sucrose formulation that closely resemble the nowadays human diet. Rhubarb supplementation is sufficient to prevent all the metabolic disorders induced by the HFHS diet. Therefore, we propose that there is a close relationship between Reg3γ and *Akkermansia muciniphila* and that this reciprocal interaction participates in the metabolic improvements in response to rhubarb supplementation.

## Supplementary Materials

The following are available online at https://www.mdpi.com/2072-6643/12/10/2932/s1, Supplementary Materials: Supplementary Table S1 Primer sequences.
