# Supplementary material for: Rhubarb Supplementation Prevents Diet-Induced Obesity and Diabetes in Association with Increased Akkermansia muciniphila in Mice
Source: Nutrients. 2020 Sep 24;12(10):2932. doi: 10.3390/nu12102932 (PMC7601677; doi:10.3390/nu12102932)
Supplement: Supplementary file 1 [file nutrients-12-02932-s001.pdf]

Supplemental table 1: Primers used for qPCR amplification

Primers		Sequences
Ang4	Forward	CTCTGGCTCAGAATGTAAGGTACGA
	Reverse	GAAATCTTTAAAGGCTCGGTACCC
Itgax	Forward	CAAAATCTCCAACCCATGCT
	Reverse	TGTGGTCAGCTCCACAGTTC
Cd163	Forward	GGCAACAAATACGTGGCTCT
	Reverse	ATGGGATTTCTCCTCCAACC
Defa	Forward	GGTGATCATCAGACCCCAGCATCAGT
	Reverse	AAGAGACTAAAACTGAGGAGCAGC
Adgre1	Forward	TGACAACCAGACGGCTTGTG
	Reverse	CAGGCGAGGAAAAGATAGTGT
Il1b	Forward	TCGCTCAGGGTCACAAGAAA
	Reverse	CATCAGAGGCAAGGAGGAAAAC
IL10	Forward	GGACAACATACTGCTAACCGAC
	Reverse	AAAATCACTCTTCACCTGCTCG
Intectin	Forward	GTTGCCCCTGATTCTGCTGG
	Reverse	GCACTATTGCAGAGGTCCGT
Ifng	Forward	TTCTTCAGCAACAGCAAGGC
	Reverse	ACTCCTTTTCCGCTTCCTGA
Lbp	Forward	GTCCTGGGAATCTGTCCTTG
	Reverse	CCGGTAACCTTGCTGTTGTT
Lys1	Forward	GCCAAGGTCTACAATCGTTGTGAGTTG
	Reverse	CAGTCAGCCAGCTTGACACCACG
Ccl2	Forward	GCAGTTAACGCCCCACTCA
	Reverse	CCCAGCCTACTCATTGGGATCA
Pla2g2	Forward	AAGGATCCCCCAAGGATGCCAC
	Reverse	CAGCCGTTTCTGACAGGAGTTCTGG
Reg3y	Forward	TTCCTGTCCTCCATGATCAAA
	Reverse	CATCCACCTCTGTTGGGTTC
Rpl19	Forward	GAAGGTCAAAGGGAATGTGTTCA
	Reverse	CCTGTTGCTCACTTGT
Tnf	Forward	TCGAGTGACAAGCCTGTAGCC
	Reverse	TTGAGATCCATGCCGTTGG
